# LB2. Safety and Efficacy of Combination SARS-CoV-2 Monoclonal Neutralizing Antibodies (mAb) BRII-196 and BRII-198 in Non-Hospitalized COVID-19 Patients

**DOI:** 10.1093/ofid/ofab466.1643

**Published:** 2021-12-04

**Authors:** Teresa H Evering, Mark Giganti, Kara W Chew, Michael Hughes, Carlee Moser, David Alain Wohl, Judith Currier, Joseph J Eron, Arzhang Javan, David A Margolis, Qing Zhu, Ulises D’Andrea, Keila Hoover, Bharat R Mocherla, Courtney Fletcher, Jonathan Li, Davey Smith, Eric Daar

**Affiliations:** 1 Weill Cornell Medicine, New York, NY; 2 Harvard T.H. Chan School of Public Health, Boston, Massachusetts; 3 David Geffen School of Medicine at UCLA; 4 UNC School of Medicine, Chapel Hill, NC; 5 David Geffen School of Medicine at University of California, Los Angeles, Los Angeles, California; 6 University of North Carolina at Chapel Hill, Chapel Hill, North Carolina; 7 National Institute of Health, Bethesda, Maryland; 8 Brii Biosciences, Chapel Hill, North Carolina; 9 Instituto Medico Rio Cuarto, Rio Cuarto, Cordoba, Argentina; 10 Miami Clinical Research, Miami, Florida; 11 Las Vegas Medical Research, Las Vegas, Nevada; 12 University of Nebraska, Omaha, Nebraska; 13 Brigham & Womens Hospital, Boston, Massachusetts; 14 University of California, San Diego, San Diego, California

## Abstract

**Background:**

SARS-CoV-2 continues to spread and the development of safe and effective therapeutics for the prevention of severe disease remains a priority. BRII-196 and BRII-198 are non-competing anti-SARS-CoV-2 mAbs with YTE triple amino acid substitution in Fc to extend half-life and reduce receptor binding, that are being studied for treatment of COVID-19 in the ACTIV-2 Trial, sponsored by NIAID and led by ACTG.

**Methods:**

ACTIV-2 evaluates safety/efficacy of investigational agents for treatment of non-hospitalized adults with mild-moderate COVID-19 under a randomized, blinded, controlled adaptive platform. BRII-196/BRII-198 (1000 mg each) as a single dose given as sequential infusions, or placebo to those at high risk of clinical progression (i.e., age ≥ 60 years or presence of other medical conditions) within 10 days of symptom onset and positive test for SARS-CoV-2. The primary endpoint was hospitalization and/or death through day 28. We report Phase 3 BRII-196/BRII-198 trial results per DSMB recommendation following an interim analysis.

**Results:**

Between January and July 2021, 837 participants (418 active, 419 placebo) from sites in the US (66%), Brazil, South Africa, Mexico, Argentina and the Philippines were randomized and received study product at time of emerging variants. Median age 49 years (Q1, Q3: 39, 58), 51% female, 17% Black/African-American and 49% Hispanic/Latino, with median 6 days from symptom onset. At interim analysis 71% and 97% had a day 28 and 7 visit, respectively. For all available data at interim review, BRII-196/BRII-198 compared to placebo had fewer hospitalizations (12 vs. 45) and deaths (1 vs. 9). At day 28 of follow-up, there was an estimated 78% reduction in hospitalization and/or death (2.4 vs. 11.1%), relative risk 0.22 (95% CI: 0.05, 0.86), P=0.00001 (nominal one-sided). Grade 3 or higher adverse events (AEs) were observed less frequently among BRII-196/BRII-198 participants than placebo (3.8% vs. 13.4%) with no severe infusion reactions or drug related serious AEs.

**Conclusion:**

BRII-196/BRII-198 was safe, well-tolerated, and demonstrated significant reduction compared to placebo in the risk of hospitalization and/or death among adults with mild-moderate COVID-19 at high risk for progression to severe disease.

**Disclosures:**

**Kara W. Chew, MD, MS**, Amgen (Individual(s) Involved: Self): Grant/Research Support; Merck Sharp & Dohme (Individual(s) Involved: Self): Grant/Research Support **David Alain Wohl, MD**, Gilead Sciences (Individual(s) Involved: Self): Advisor or Review Panel member, Consultant, Research Grant or Support, Scientific Research Study Investigator; Janssen (Individual(s) Involved: Self): Advisor or Review Panel member; Merck (Individual(s) Involved: Self): Advisor or Review Panel member, Research Grant or Support; ViiV (Individual(s) Involved: Self): Advisor or Review Panel member, Research Grant or Support **Joseph J. Eron, MD**, **Gilead Sciences** (Consultant, Research Grant or Support)**Janssen** (Consultant, Research Grant or Support)**Merck** (Consultant)**ViiV** (Consultant, Research Grant or Support) **David A. Margolis, MD MPH**, **Brii Biosciences** (Employee) **Courtney Fletcher, Pharm.D.**, **National Institute of Allergy and Infectious Diseases, NIH** (Grant/Research Support) **Davey Smith, M.D.**, **Linear Therapies, Matrix Biomed, Bayer** (Consultant, Shareholder) **Eric Daar**, **Gilead** (Consultant, Grant/Research Support)**Merck** (Consultant)**ViiV** (Consultant, Grant/Research Support)

